# Relevance of clinical ethics support services in specialized outpatient palliative care teams and inpatient hospices

**DOI:** 10.1186/s12904-026-02149-2

**Published:** 2026-05-23

**Authors:** Carola Seifart, Marcel Engelhardt, Irene Portig, Timo Sauer, Christian Volberg

**Affiliations:** 1 Marburg University, Faculty of Medicine, Institute for Applied and Clinical Ethics, Marburg, Germany; 2 Marburg University, Faculty of Medicine, Department of Anesthesiology & Intensive Care Medicine, Baldingerstraße, Marburg, 35043 Germany; 3 Goethe University of Frankfurt, Dr. Senckenberg’s Institut for the History of Medicine and Medical Ethics, Frankfurt / Main, Germany

**Keywords:** Ethics consultation service, Palliative care, Hospice, Specialized outpatient palliative care service, Clinical ethics, Ethical conflict, Caregiver burdens

## Abstract

**Background and objectives:**

Clinical ethics support services (CES) may support palliative care teams in ethical conflicts. However, it is not known to what extent these services are available, used and how their relevance is perceived by the teams. The study focuses on the accessibility and evaluation of ethics consultations in specialized outpatient palliative care teams and inpatient hospices.

**Methods:**

This study conducted a cross-sectional 21-item anonymous survey among teams of registered specialized outpatient palliative care services (*n* = 304) and inpatient hospices (*n* = 260) in Germany concerning availability, utilization and evaluation of clinical ethics support services.

**Results:**

A total of 175 (58%) specialized outpatient palliative care teams and 102 (39%) inpatient hospice teams responded to the questionnaire; of these two-thirds have access to CES. A number of ethical challenges were reported in these settings, many of which are perceived as burdensome. However, satisfaction with existing services is rather moderate and teams report fewer than five consultations per year. Nevertheless, ethics consultation is regarded as being highly useful, with positive effects reported, including enhanced confidence in decision-making processes and reduced burden for team members. Interestingly, team members with formal training in clinical ethics had a significant positive impact on the perception of CES and the frequency of consultations.

**Conclusion:**

Although specialized outpatient palliative care and inpatient hospice teams reported facing a number of burdensome ethical challenges, ethics consultations fall short of accessibility to CES. This discrepancy can be resolved by structured training of team members in clinical ethics to increase the level of awareness and utilization of CES in specialized outpatient palliative care and inpatient hospices.

**Supplementary Information:**

The online version contains supplementary material available at 10.1186/s12904-026-02149-2.

## Introduction

Taking care of palliative patients leads to a multitude of challenges, potential problems and conflicts. Part of these challenges include ethical dilemma situations or possible ethical conflicts when decisions must be made in clinical care. These often arise in association with the question of when, to whom and in what form bad news should be delivered [1–3], problems concerning place of care and death [2], justice in access to palliative care [2], values laden conflicts between healthcare professionals, the patient and their relatives or any combination [4, 5], decisions about continuation or discontinuation of treatment, especially in end-of-life decisions [3, 6–9]. Dealing with and solving such challenges ideally requires a deliberation process [6, 10]. If this is not effective, negative consequences for patients, relatives and healthcare providers may result, such as suboptimal patient centered-care, higher level of moral distress or intensified communication conflicts [11, 12].

Therefore, for several decades, the method of ethical case consultation has been established to support deliberation processes in medical decision-making with ethical challenges. Ethics (case) consultation is a process that could be “provided by an individual consultant, team or committee to address the ethical issues involved in a specific clinical case“ [13]. Ethical challenges can be understood as uncertainties or conflicts related to moral values or norms relevant to the care of a patient in clinical practice [14]. The central task of ethics consultation is to improve the process and outcomes of ethical challenges that arise in the context of patient care. Ethics consultation can help identify, analyze and contribute to the resolution of these challenges [13]. Ethics consultation is usually provided through various organizational forms for clinical ethics support services, whereby they are very well established in hospitals and are used to varying degrees, depending on the respective clinical areas [15, 16]. In 2014, the Council of Europe published the “Guidelines for the decision-making process regarding medical treatment in end-of-life situations” via its Committee on Bioethics. This guideline recommends that clinical ethics support services should be involved in difficult and uncertain end-of-life situations [17]. However, information about implementation, utilization, challenges and users’ evaluation towards clinical ethics support services in the area of palliative medicine is missing.

In Germany, there are two models of palliative care which can be divided into general and specialized care. General care is provided by family doctors and in general medical treatment facilities (e.g., oncology wards) for patients who do not have a particularly severe symptom burden and do not require excessive medical care. The specialized care of patients with non-curable diseases and a high symptom burden is divided into three areas, which are, however, in contact with each other. Acute medical problems (e.g. acute exacerbation of distressing symptoms) are treated in inpatient palliative care units attached to a hospital. Outpatient care is provided with the help of specialized outpatient palliative care services either in the home environment, so that care can be ensured by relatives and care services, or in an inpatient hospice if a high level of care is required and home care is not possible [18, 19]. In addition, there are other palliative care facilities, such as day hospices or palliative care day clinics, some of which are included in palliative care networks [18].

For the constitution, organization and membership of clinical ethics support services there are no formally binding regulations or guidelines in Germany. Therefore, clinical ethics support services could be organized and staffed in different ways, particularly in outpatient settings [20]. A central task of a clinical ethics support service is to offer and conduct ethics consultations.

The professional German association “Akademie für Ethik in der Medizin” (Academy for Ethics in Medicine) recommends formal training in clinical ethics for ethics consultants. However, this training is not a binding prerequisite for members of clinical ethics support services or for providing ethics consultations [21].

Specialized outpatient palliative care services and inpatient hospices were selected as typical examples of palliative care facilities to investigate whether ethics consultation services are established, their utilization and their appraisal in a nationwide survey. Furthermore, the study aimed to evaluate which ethical challenges and topics are perceived as relevant for ethics consultation in these settings.

## Methods

### Research question & study design

The research question aimed to obtain information about the implementation, use, topics, challenges, advantages, obstacles, and user evaluation of ethical counseling in outpatient palliative care. To this end, a national cross-sectional survey was designed among specialized outpatient palliative care services and inpatient hospices in Germany. This survey was conducted using a specially developed anonymous questionnaire.

### Design of questionnaire

An interdisciplinary team with expertise in the development of questionnaires (CS, CV, IP, TS), consisting of palliative care physicians (CV), nurses (TS) and clinical ethics experts (TS, CS, IP) developed a questionnaire with 21 items. Initially, the questionnaire was presented to three nurses and two physicians from the field of palliative care to check for comprehensibility and consistency. As a result, understanding was generally good, a few formulations were adapted for better intelligibility, and a definition of ethic consultation was included in the questionnaire. A translated questionnaire can be found in the supplementary material.

In addition to demographic variables, the questionnaire touched on the availability and use of ethics consultation, the frequency and usual procedure for consultation and the evaluation of ethics consultation. Possible positive effects and obstacles were also surveyed. If ethical case consultation was not used in the respective institution, we asked for barriers to utilization. To avoid misunderstandings, respondents were provided with a definition of ethics consultation (“*Ethics case consultation is a process for addressing and resolving ethically relevant decision-making challenges in healthcare practice*,* usually in the format of a facilitated discussion with an ethics consultant*”).

The questionnaire contained ‘yes’ or ‘no’ questions, questions on categorization with single and multiple choice and rating scales with an 11-point Likert scale (0–10). On the latter, 0 meant “not at all” and 10 “extremely”. A number of questions featured an option to give additional free text answers.

Next, participants were asked to indicate whether and how often certain topics had prompted ethics consultations. Participants were able to choose from a list of 17 possible topics and indicate whether these were frequently, occasionally or never the focus of ethics consultation. There was also the option of specifying additional topics in free text.

### Ethical approval

The study was approved by the institutional review board prior to initiation (Faculty of Medicine, University of Marburg, Ref.: #66/21).

### Recruitment & participants

The questionnaire was sent to the management unit of each institution of all specialized outpatient palliative care services (*n* = 304) and inpatient hospices (*n* = 260) listed on the homepage of the German Society for Palliative Medicine (DGP) in Germany [22]. Since the use of the address list on this homepage allowed us to assume that this was a national census, no separate inclusion criteria had to be defined.

The accompanying cover letter explicitly invited participants to answer the survey together within the team. It was also requested to indicate who completed the questionnaire (see Demographics, Table 1). The survey was anonymous, and the questionnaire could be returned to the study team using a prepaid envelope. All responses received in the period from April to December 2021 were included. No reminder letter was sent.

### Data analysis

The percentage of institutions that stated that certain situations frequently, occasionally or never triggered ethics consultations was given as a percentage of the number of responding institutions. The free-text responses were recorded accordingly.

Statistical analysis was done descriptively using Microsoft Excel Version 16.6 and SPSS (IBM Corp., Armonk, NY, USA, Version 29, 2022). Statistical analysis were based on theoretical considerations. No random statistical analyses were performed. Pearson´s correlation was used to calculate a relationship between two variables. Regarding the question of which occasions represent the greatest burden for the team, the participants were asked to indicate the three most stressful occasions. The aforementioned topics for perceived burdens were evaluated accordingly. Because these three occasions were not hierarchized, each topic mentioned was included equally in the evaluation. The results are given as a percentage of the total number of responses to the corresponding question.

Where there were free-text responses, these were taken into account as data points and mentioned in the results (e.g. Topics & Burdens). No formal qualitative analysis was performed.

## Results

### Demographics

Over the survey period, the questionnaires were returned by 175 (58%) specialized outpatient palliative care teams and 102 (39%) inpatient hospices. Of the questionnaires returned from specialized outpatient palliative care teams, one questionnaire could not be included in the analysis because more than half of the answers were not given. It was noticeable that the responses differed between specialized outpatient palliative care teams and inpatient hospices in terms of their professional background: While in the specialized outpatient palliative care teams 43% of respondents were physicians, in inpatient hospices almost all questionnaires were answered by nurses or management staff. Sociodemographic data are presented in Table 1.

**Table 1:** Demographics of participating institutions

**Demographics**			
		**Hospice**	**Outpatient**
Queries returned (response rate)		102 (39%)	175 (58%)
Employee filling the questionnaire		*N* = 98	*N* = 172
Team; together		16 (16%)	33 (19%2)
Nursing staff		36 (36%)	38 (22%)
Physician staff		1 (1%1)	74 (43%)
Others		51 (52%)	32 (19%)
Number of patients cared for per year		*N* = 101	*N* = 172
1–50	1-100	1 (1%)	6 (3.5%)
51–100	101–200	26 (26%)	17 (9.9%)
101–150	201–300	50 (50%)	43 (25.0%)
151–200	301–400	17 (17%)	34 (19.8%)
> 200	> 400	7 (7%)	72 (41.9%)
Number of employees		*N* = 101	*N* = 170
1–10		0%	46 (27%)
11–20		12 (12%)	70 (41%)
21–30		34 (34%)	23 (14%)
31–40		21 (21%)	10 (6%)
> 40		34 (34%)	21 (12%)
Residential area		*N* = 101	*N* = 172
Metropolis (> 1 Mio. inhabitants)		6 (6%)	17 (10%)
Large city (> 100.000 inhabitants)		36 (36%)	63 37%
Medium-sized city (< 100.000 inhabitants)		32 (32%)	49 (29%)
Small town, rural area (< 20.000 inhabitants)		27 (27%)	43 (25%)

The results show that in specialized outpatient palliative care teams and inpatient hospices clinical ethics support services and the method of ethics (case) consultations are generally known: 85% of respondents from specialized outpatient palliative care services and from inpatient hospices stated that they were aware of ethics consultation. Slightly fewer have access to clinical ethics support services (74% outpatient; 78% hospices). Around three quarters of participants stated that they had already taken part in an ethics consultation (77% outpatient; 76% hospices).

### Utilization

When asked whether ethics (case) consultation was used in everyday clinical practice at their institution, around two-thirds agreed (67% outpatient; 69% hospices), while one-third did not use ethics consultation (33% outpatient; 29% hospices).

The majority of participants in both groups stated that less than five ethics consultations were organized per year. Estimated numbers can be found in Table 2. The numbers of ethics consultations increase predominantly with the number of patients treated in specialized outpatient palliative care services. However, in inpatient hospices the number of ethics consultations was independent of the numbers of patients treated per year. When a team member had formal training in clinical ethics, respondents reported more ethics case consultations: 40.8% of inpatient hospices and 33.9% of specialized outpatient palliative care teams stated to have 6 or more consultations per year, compared to only 28.8% (outpatient) and 23.1% (hospice) of teams without internal trained ethics consultant (compare Table 2).

**Table 2:** Framework of ethics consultation

	**Hospice**	**Outpatient**
Do you have access to ethics consultation service?	*N* = 102	*N* = 174
Yes	80 (78%)	129 (74%)
No	10 (10%)	25 (14%)
Unsure	12 (12%)	20 (11%)
Are there trained ethics consultants in your team?	*N* = 68	*N* = 112
Yes	30 (42%)	66 (59%)
0–5 consultation/year	59%	66%
6–10 consultation/year	26%	23%
> 10 consultation/year	15%	11%
No	36 (53%)	39% (44)
0–5 consultation/year	70.6%	77.0%
6–10 consultation/year	20.6%	15.4%
> 10 consultation/year	8.8%	7.7%
Uncertain	2 (3%)	2 (2%)
How do you usually deal with ethical issues (multiple answers)?	*N* = 70	*N* = 113
Internal meeting rounds	58 (83%)	81 (72%)
Internal trained ethics consultant	19 (27%)	51 (45%)
Own Ethics Committee	19 (27%)	36 (32%)
Goal of care discussions	13 (19%)	47 (43%)
External trained ethic consultant	15 (21%)	6 (5%)
External Ethics Committee	5 (7%)	14 (12%)
Other (round table etc.)	9 (13%)	14 (12%)

The participants were also asked how they deal with ethical issues in everyday clinical practice. The most common answer was internal meetings, followed by meetings with an internal ethics consultant and goal of care discussions. Results can be found in Table 2. Approximately one-third (28%) of inpatient hospices and one-fifth (17%) of specialized outpatient palliative care services reported using ethics consultation from external facilities.

### Topics & burdens

Participants were asked which situations resulted in an ethics case consultation and how often this happened. The most frequently given reasons for ethics case consultations are treatment limitation, artificial nutrition, identification of patients’ preferences and conflicts between relatives and patients or the team. There were only slight differences between the responses from members of outpatient palliative care teams and hospices. Five hospice teams added palliative sedation and two admission regulations to their free-text responses. Results are listed in Table 3.

**Table 3:** Ethical conflict situations

	Total (*N*/%)	Frequently	Occasionally	Never
Specialized outpatient palliative care services				
Treatment Limitation	112 (93%)	49 (44%)	55 (49%)	8 (7%)
Artificial Nutrition/PEG	107 (89%)	42 (38%)	49 (46%)	12 (11%)
Identification of Presumed Patient Preferences	110 (85%)	46 (43%)	52 (47%)	16 (15%)
Execution of Patient Living Will	109 (76%)	25 (23%)	58 (53%)	26 (24%)
Ethical Conflict between Relatives and Team	111 (85%)	24 (22%)	70 (63%)	17 (15%)
Ethical Conflicts between Relatives and Patient	110 (87%)	23 (21%)	73 (66%)	14 (13%)
Expressions of Intent in Dementia Patients	109 (81%)	16 (15%)	72 (66%)	21 (19%)
Refraining from Nutrition	113 (77%)	15 (13%)	72 (64%)	26 (23%)
Request for assistance in Suicide	111 (74%)	14 (13%)	68 (61%)	29 (26%)
Privacy Rights of Residents/Patients	106 (37%)	14 (13%)	25 (24%)	67 (63%)
The following were mentioned less frequently overall: Ethical conflicts within the treatment/care team; Capacity to consent of residents/patients; Interreligious or intercultural problems; Privacy Rights of Residents/Patients; Violence against persons or property; Compulsory treatment of residents/patients; Problems with sexuality; Measures that deprive individuals of their liberty (e.g., restraint); Sexual assault				
Inpatient Hospices				
Identification of Presumed Patient Preferences	68 (79%)	7 (10%)	50 (74%)	11 (16%)
Treatment Limitation	67 (78%)	6 (9%)	50 (74%)	11 (16%)
Ethical Conflicts between Relatives and Patient	68 (79%)	5 (7%)	50 (74%)	13 (19%)
Ethical Conflict between Relatives and Team	68 (79%)	4 (6%)	50 (74%)	14 (21%)
Artificial Nutrition/PEG	67 (78%)	9 (13%)	43 (64%)	15 (22%)
Refuse of Medical or Nursing Assistance	66 (59%)	5 (7%)	34 (52%)	27 (41%)
Request for assistance in Suicide	68 (57%)	5 (7%)	34 (52%)	29 (43%)
Execution of Patient Living Will	67 (55%)	4 (6%)	33 (49%)	30 (45%)
Ability to Consent by Patients	68 (54%)	5 (7%)	32 (47%)	31 (46%)
Privacy Rights of Residents/Patients	66 (47%)	4 (6%)	27 (41%)	35 (53%)
The following were mentioned less frequently overall: Capacity to consent of residents/patients; Expressions of will in residents/patients with dementia; Privacy Rights of Residents/Patients; Interreligious or intercultural problems; Violence against persons or property; Problems with sexuality; Sexual assault; Measures that deprive individuals of their liberty (e.g., restraint); Compulsory treatment of residents/patients				

In addition, the participants were asked to write down the three situations that are most burdensome for the team. In outpatient palliative care services, the request for assisted suicide was mentioned most frequently, followed by treatment limitations and the problem of artificial nutrition. In hospices, conflicts between relatives and patients and within the team and treatment limitation were first mentioned. Hospice teams added in their free-text responses: palliative sedation (6), admission regulations (2), forced patient transfers (e.g., to another institution) (1) and return to home care (1). Specialized outpatient palliative care teams also indicated palliative sedation (4) and rejection of pain relief (1) in the free texts. Treatment limitation (outpatient and hospice) and conflicts between relatives and patients (hospices) are stressful situations that are also mentioned among the most frequent reasons for requests for ethics consultation.

However, this does not apply to all burdensome situations. Although the burden caused by requests for assisted suicide are very high in the outpatient palliative care situation, they are not among the most frequent reasons for ethics consultations. The same applies to the determination of presumed patient preferences in hospices. The most challenging ethical conflict situations are listed in Table 4.

**Table 4:** Most burdensome ethical conflict situations

**Specialized outpatient palliative care teams**	% of 304 given answers / 110 responder	
Request for assistance in Suicide	15%	47
Treatment Limitation	14%	44
Artificial Nutrition/PEG	12%	37
Ethical Conflicts between Relatives and Patient	12%	37
Ethical Conflict between Relatives and Team	11%	34
Identification of Presumed Patient Preferences	8%	24
Refuse of Medical or Nursing Assistance	6%	18
Interreligious or Intercultural Conflicts	5%	14
Execution of Patient Living Will	4%	12
Expressions of Intent in Dementia Patients	4%	11
Refraining from Nutrition	3%	9
Ethical Conflicts within the Team	3%	9
Ability to Consent by Patients	2%	5
Privacy Rights of Residents/Patients	0,3%	1
Violence against Individuals or Objects	0,3%	1
Forced Treatment of Patients	0,3%	1
Sexual Problems	0%	0

Inpatient Hospices	% of 187 given answers / 69 responder	
Ethical Conflicts between Relatives and Patient	14%	27
Ethical Conflict between Relatives and Team	11%	21
Treatment Limitation	11%	21
Ethical Conflicts within the Team	11%	20
Request for assistance in Suicide	10%	18
Artificial Nutrition/PEG	10%	18
Refuse of Medical or Nursing Assistance	10%	18
Identification of Presumed Patient Preferences	6%	11
Refraining from Nutrition	6%	11
Forced Treatment of Patients	2%	4
Expressions of Intent in Dementia Patients	2%	4
Interreligious or Intercultural Conflicts	2%	3
Execution of Patient Living Will	2%	3
Ability to Consent by Patients	2%	3
Violence against Individuals or Objects	2%	3
Privacy Rights of Residents/Patients	1%	2
Sexual Problems	0,5%	1

### User´s evaluation

**Fig. 1:**
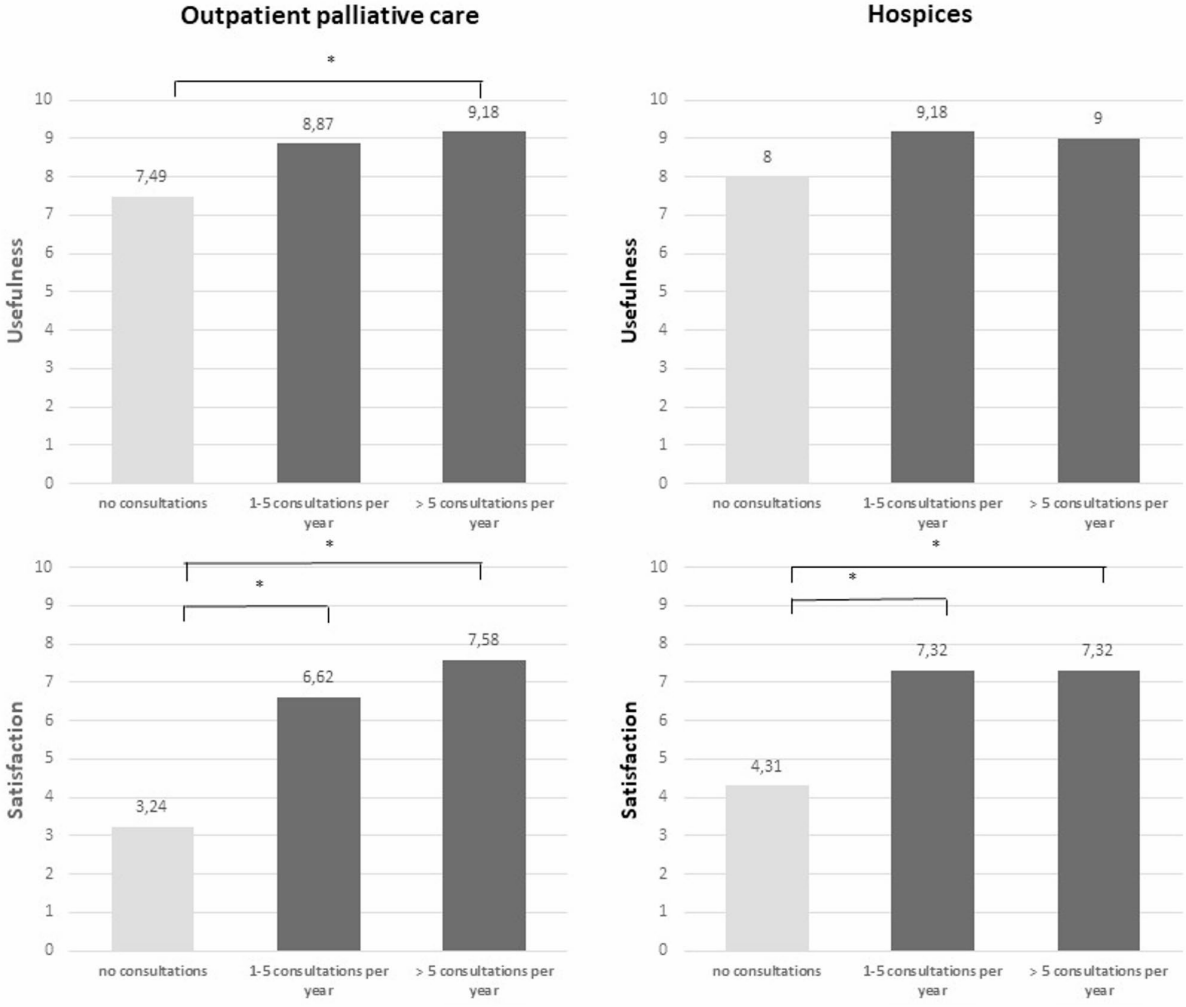
Correlation between the frequency of use of ethics case consultation and the assessment of general usefulness and satisfaction with clinical ethics support service available. The ratio between the number of ethics consultations per year and the evaluation of the usefulness of ethics consulting is shown. The more ethics consultations there are, the higher the assessment of the usefulness of ethics consulting. (*p < 0.01)

With regard to the evaluation of their experiences with clinical ethics support services, we asked whether the respondents consider ethics consultation to be useful in general, how high they rate the need for ethics consultation in their daily work and how satisfied they are with the options for ethics consultation that are available to them. There were no significant differences in the evaluation of outpatient palliative care services and hospices: ethics consultation service is generally rated as very useful (mean+-SD 8.49+-2.82 outpatient; 8.78+-1.45 hospices (Likert 1–10)), whereby a medium need for ethics consultation is seen (5.5+-2.70 outpatient; 5.21+-3.40 hospices (Likert 1–10)) and satisfaction with the available clinical ethics support services falls short to the reported usefulness (5.96+-3.21 outpatient; 6.72+-3.15 hospices (Likert 1–10)). Interestingly, the assessment of ethics consultation as satisfactory was strongly correlated to the frequency of use (outpatient: pearson’s correlation *r* = 0.62, *p* < 0.01; hospice *r* = 0.61; *p* < 0.01). Also, the assessment of ethics consultations as useful showed a moderate positive correlation with the frequency of use (outpatient: pearson’s correlation *r* = 0.38; *p* = 0.011; hospice *r* = 0.39, *p* < 0.01). Results are shown in Fig. 1.

**Fig. 2:**
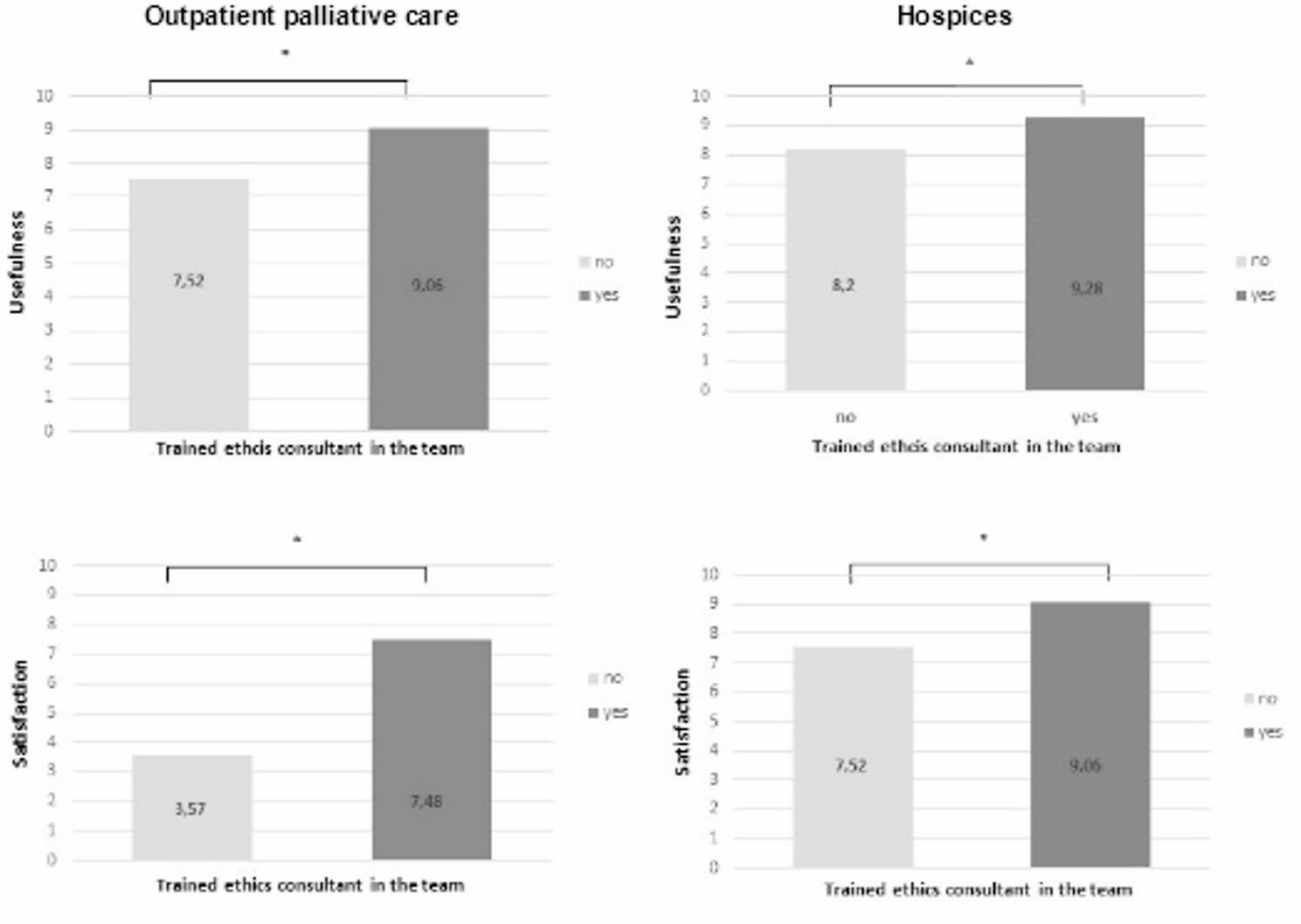
Correlation between trained ethics consultants with the assessment of the general usefulness of ethics consultation and satisfaction with clinical ethics support service available. (* *p* < 0.01)

We then examined how respondents solve ethical questions in everyday life. The statistical analysis, which examined whether the type of dealing with ethical challenges was associated with the evaluation of ethics support services, revealed a positive trend for internal discussions in both settings, with a positive correlation between usefulness in inpatient hospices and satisfaction in specialized outpatient palliative care services, respectively. If trained ethics consultants are part of the teams in specialized outpatient palliative care teams, both the usefulness of ethics consultation in general (pearson’s correlation *r* = 0.35, *p* < 0.01) and satisfaction with ethics consultation services available are rated higher (outpatient: pearson’s correlation *r* = 0.55, *p* < 0.01). In hospices this effect was also seen but far less pronounced for usefulness (*r* = 0.11, *p* < 0.01) and satisfaction (*r* = 0.21; *p* < 0.01). Interestingly, if an internal ethics consultant is present and meeting rounds are used, a significant effect on satisfaction was seen in specialized outpatient palliative care services (*r* = 0.51; *p* < 0.01) and to lesser extent also in hospices (pearson’s correlation *r* = 0.3; *p* < 0.01). Results are presented in Fig. 2.

Regarding perception of ethics counselling as generally useful there was no apparent difference whether the questionnaire was answered by nursing staff, the whole team, or others working either in an inpatient hospice or in specialized outpatient palliative care services. Given the relatively small size of the respective groups, nursing staff seem to be more satisfied with ethics support services than the team as a whole or others, while this observation applied to individuals in specialized outpatient palliative care services who identified themselves as part of the management team.

### Benefits & barriers

The respondents were asked if they experienced positive effects through ethics consultations and if they observed barriers to realization. In both, specialized outpatient palliative care teams and inpatient hospices the relief of involved participants and the enhancement of trust in challenging decisions ranked first, followed by precise identification of the problem and transparency of decision making.

The barriers relate to logistical issues, the lack of awareness regarding ethics consultations, or a lack of acceptance. Perceived benefits and barriers mentioned are listed in Table 5.

**Table 5:** Benefits and barriers to outpatient ethics consultation services

Perceived benefits of outpatient ECS	Inpatient Hospices *N* = 70	Specialized outpatient palliative care *N* = 114
Relief for involved participants	77%	63%
Enhancement of trust in challenging decisions	61%	58%
Precise identification of problems	50%	47%
Transparency of decision making	41%	36%
Better implementation of preferences	33%	25%
Improved communication	30%	27%
Increased motivation in addressing issues	23%	8%
Reassurance of the decisions accuracy	20%	20%
Successfully resolved conflicts	19%	19%
Respectful interactions with one another	16%	14%
Decisions are received more positively	11%	6%
There are no positive effects	0%	0%

Barriers to ECS	*N* = 101	*N* = 165
Arranging appointments with involved parties	30%	44%
ECS remains unknown	24%	35%
Minimal usage despite identified necessity	18%	33%
Meeting cannot be integrated into work routine	12%	23%
Limited knowledge about responsibilities of ECS	18%	19%
Too much effort	5%	15%
Low acceptance by relatives	11%	11%
No challenges to address	31%	10%
Lack of acceptance from attending physicians	6%	8%
Implementation of the recommendations of ECS	4%	7%
ECS is not supported in the institution	0%	1%

## Discussion

The study shows that specialized outpatient palliative care teams and inpatient hospice teams reported facing a number of burdensome ethical challenges. Ethics support services are well known and widely accessible, are rated very highly overall, whereby user evaluation is linked to usage. Nevertheless, the number of reported ethics consultation is rather low. A number of benefits of and barriers to ethics consultations were mentioned, whereas in teams that included a trained ethics consultant, the frequency of ethics consultations was higher and the evaluation of ethics consultations in general, as well as the available services, were significantly better.

Various guidelines explicitly recommending ethics consultations for uncertain or difficult situations in palliative care [17, 18, 23]. However, to our knowledge, this is the first study to address the use and evaluation of ethics consultation in the field of palliative care.

With the exception of the request for assisted suicide and palliative sedation, the named reasons for ethics consultations do not significantly differ from those that are also common in other medical fields [24, 25]. To a certain extent, this is surprising, since in palliative medicine at least, there is no expectation of dilemma regarding the general question of curative treatment intention. However, acknowledging that a disease isn’t curable, palliative care professionals still face many treatment decisions. This is emphasized by the study, which identified a series of situations that were considered as ethical challenges and were also perceived as burdensome by the teams.

Around three quarters of palliative care nurses report that ethical dilemmas occur on a monthly or even weekly basis [26]. The PALCOM study showed one third of patients with advanced cancer face ethical dilemmas [27]. In the present study, a number of ethical challenges, including treatment limitation, artificial nutrition and ethical conflicts between relatives and patients, were named to occur often by the majority of palliative care teams. Therefore, it can be assumed that ethical challenges play a relevant role in palliative care in terms of their significance and frequency. Although the participants rated ethics consultation generally as a very useful tool corresponding to results from other medical areas [28–30], 70% of institutions reported having only 0–5 consultations per year. Even though it is impossible to determine exactly how many ethical challenges arise in a palliative care team over the course of a year, it can be assumed that ethics consultation services are obviously underutilized. Underutilization of ethics consultation is not a new observation and therefore discussed in others areas as well [31, 32]. Various explanations have been put forward: delaying decision making or the feeling that health care professionals should decide themselves, varied training of ethics consultants or lack of knowledge of the procedure [33, 34]. While this study supports these findings at large, it adds especially administrative barriers and knowledge gaps. Interestingly, the disparity between low utilization and observed need was explicitly mentioned as a barrier by the respondent teams. Nonetheless, about a third of inpatient hospices teams reported under barriers, that there were “no problems”. This answer could point to an additional aspect: Considering that palliative care is regarded a “deeply ethical practice”, the accompanying professional ethos may imply that ethical challenges should be solved without external help. 

This could also explain the rather low perceived need for ethics consultations. Another possible reason is that palliative health care professionals and clinical ethics consultants have overlapping competencies within the area of communication [35, 36]. Therefore, the support of ethics consultants might be considered unnecessary. However, this overlooks the fact that ethics consultants provide support that goes far beyond communication. Among other aspects, ethics consultants bring a necessary “outside perspective” in situations where decisions are difficult, helping to identify underlying values and potential biases [35]. Moreover, specialized outpatient palliative care teams may be consulted to help addressing particularly challenging situations, such as requests for assisted dying. Last, but not least, even if a communication problem within the team seems solvable, uncertainty and moral distress, which are potential effects of ethical challenges, may still remain. Real underutilization can result in a lack of necessary deliberation process in situations of difficult decision making. Margaret Urban Walker proposed that clinical ethicists are architects of “moral spaces” serving to create resonance rooms for morally difficult situations [37]. This concept was considered to be highly meaningful and indicative [38] which expresses the fundamental importance of ethics consultation, as demonstrated, for example, during the COVID-19 crisis [39, 40]. The primary objective of ethics case consultations is to support decision-making in ethically challenging situations. This, in turn, results in a greater likelihood of finding the “best” solution together [41, 42]. By creating opportunity for this certainty, moral spaces are important to support the quality of patient care [43, 44]. In addition, ethical case consultations support the teams with regard to moral distress and burdens [45].

The responses in this study confirm that the most important perceived benefit of ethics consultation is relief. This is consistent with the observation that ethical counseling creates a healthier working climate for employees [44, 46]. Therefore, it seems important to address the barriers to ethics consultations in order to improve access to these “moral spaces” in palliative care, an area that is known for its challenging situation overall. Training team members in clinical ethics appears to be effective in this regard and seems to offer a remedy. The results of the present study suggest that the presence of trained ethics consultants in palliative care teams leads to a higher frequency of consultations and is associated with a positive basic attitude towards and a higher level of satisfaction with the available clinical ethics support services. The training of team members in clinical ethics has been recommended for other clinical fields and has been shown to correlate with the number of consultations [32, 47].

However, it should be borne in mind that, on the one hand, it is important for ethics consultants to assume a “neutral” or “outsider” role in the consultation process, not at least because they may be involved in advocating for patients [35, 36, 39]. On the other hand, conflicts of interest must be avoided [48]. Therefore, members of the treatment team should not be ethics consultants if they have clinical or administrative responsibility for the case under discussion. By taking these role conflicts into account, internal ethics consultants can not only meet logistical challenges but also contribute to raising awareness of ethical issues within the team.

Therefore, a better information policy and overcoming of the aforementioned logistical hurdles can both be achieved by having trained ethics consultants in the team. Furthermore, training of team members in clinical ethics raise sensitivity for ethical issues and therefore counteract underutilization, whereby help counter the burden and moral distress arising from ethical challenges in palliative care by creating “moral spaces” and a healthier work climate [38, 49].

### Limitations

When evaluating the study results, several possibilities for bias must be taken into account. In addition to the usual limitations arising from questionnaire studies, such as the influence of prior assumptions, a confirmation bias is not unlikely. Furthermore, allowing the management of each institution to choose the participants may have introduced a selection bias. Although the response rate of 39% of inpatient hospices and 58% of specialized outpatient palliative care teams is good, it is not representative. It is possible that only teams with a positive attitude towards ethics consultation responded and that the results are biased in this sense. A further weakness of the study is that no specific questions were asked about individual experiences with ethics consultation and therefore possible positive or negative experiences cannot be specifically included in the evaluations. Further studies are needed to obtain more information on the question of underutilization and to prove a reliable connection between possible barriers and their influence on the use of ethics consultation.

## Conclusion

There are a number of frequently ethical challenges associated with significant burdens for team members of specialized outpatient palliative care services and inpatient hospices. Clinical ethics support services seem to be widely accessible and may create helpful “moral spaces” to deal with these challenges.

Although clinical ethics support services were perceived very positively by team members, utilization falls short of accessibility. However, in teams that included a trained ethics consultant, the frequency of ethics consultations was higher and the evaluation of ethics consultations in general, as well as the available services, were significantly better. The integration of ethics consultants into teams or the training of team members in clinical ethics can therefore be recommended to enable the establishment of important moral spaces to support palliative care team members and reduce burdens.

## Supplementary Information

Supplementary Material 1.

Supplementary Material 2.

## Data Availability

The datasets used and analyzed in the current study are available from the corresponding author on reasonable request.
