# Supplementary material for: Relevance of clinical ethics support services in specialized outpatient palliative care teams and inpatient hospices
Source: BMC Palliat Care. 2026 May 23;25:154. doi: 10.1186/s12904-026-02149-2 (PMC13198748; doi:10.1186/s12904-026-02149-2)
Supplement: Supplementary file 1 — Supplementary Material 1. [file 12904_2026_2149_MOESM1_ESM.docx]

# Translated Questionnaire Hospice

## Questionnaire: Ethics Case Consultation in German Hospices

1. Who is completing this questionnaire?

☐ jointly as a team

☐ nursing staff

☐ physician

☐ other professional group: _______________________________

2. How many employees work in your hospice? (including contracted physicians, volunteers, etc.)

☐ 1–10

☐ 11–20

☐ 21–30

☐ 31–40

☐ >40

3. How many patients do you care for on average per year?

☐ 1–50

☐ 51–100

☐ 101–150

☐ 151–200

☐ >200

4. In which federal state is your hospice located?

___________________________________________________

What type of residential area does your catchment area correspond to?

☐ Metropolis (> 1 million inhabitants)

☐ Large city (> 100,000 inhabitants)

☐ Medium-sized city (< 100,000 inhabitants)

☐ Small town, rural area (< 20,000 inhabitants)

5. Are you familiar with ethics case consultation services?

☐ yes

☐ no

☐ unsure

6. Have you ever participated in an ethics case consultation?

☐ yes

☐ no

☐ unsure

7. Do you have the opportunity to use or request ethics case consultation?

☐ yes

☐ no

☐ unsure

8. Do you use ethics case consultation in your current professional practice?

☐ yes

☐ no

☐ unsure

If yes, how often: ☐ 0–5/year ☐ 6–10/year ☐ 11–20/year ☐ >20/year

9. If you answered “no” to question 8, please indicate the three main reasons why you do not use ethics case consultation (max. 3 answers, fewer are allowed):

☐ there is no such service available

☐ so far, we have resolved all problems without assistance

☐ using ethics case consultation is too time-consuming in daily practice

☐ ethics case consultation does not seem helpful to us

☐ the effort/benefit ratio is too poor

☐ there is resistance within the team

☐ involving external persons is not desired

☐ it is difficult to bring all parties “to the table”

☐ other: ____________________________

If you answered “no” to question 8, you can skip directly to question 16.
If you answered “yes,” please continue here (with question 10).

10. How do you address ethical issues in everyday practice?

☐ via an in-house ethics committee

☐ ethics café (open discussion forum)

☐ internal team meetings

☐ via a (supra-)regional ethics committee

☐ via a trained ethics consultant within the team

☐ via a trained ethics consultant from outside

☐ via an external ethics consultation team (e.g., via the medical association)

☐ we resolve ethical problems through discussions about therapy goals

☐ other: _____________________________________

11. Does your team include trained ethics consultants (at least AEM* Level 1)?
*AEM: Academy for Ethics in Medicine

☐ yes

☐ no

☐ unsure

If yes, how many? ____________

12. Please indicate how often (never, occasionally, often) the following ethical conflict situations have played a role in ethics case consultations in your team:

| Conflict | Never | Occasionally | Often |
| --- | --- | --- | --- |
| Limitation of therapy (e.g., ending medical treatment or transfer to hospital at end of life) | ○ | ○ | ○ |
| Artificial nutrition/PEG (e.g., initiation or discontinuation) | ○ | ○ | ○ |
| Implementation/handling of advance directives | ○ | ○ | ○ |
| Determining the presumed will of the patient | ○ | ○ | ○ |
| Patient’s capacity to consent | ○ | ○ | ○ |
| Patient’s right to privacy | ○ | ○ | ○ |
| Expressions of will in patients with dementia | ○ | ○ | ○ |
| Patient refuses medical or nursing care | ○ | ○ | ○ |
| Voluntary refusal of food and drink | ○ | ○ | ○ |
| Request for assistance with suicide | ○ | ○ | ○ |
| Forced treatment of patients | ○ | ○ | ○ |
| Deprivation of liberty measures (e.g., restraint) | ○ | ○ | ○ |
| Violence against persons or objects | ○ | ○ | ○ |
| Sexual assaults | ○ | ○ | ○ |
| Problems related to sexuality | ○ | ○ | ○ |
| Interreligious or intercultural problems | ○ | ○ | ○ |
| Ethical conflicts between relatives and caregivers | ○ | ○ | ○ |
| Ethical conflicts between relatives and the patient | ○ | ○ | ○ |
| Ethical conflicts within the treatment team | ○ | ○ | ○ |
| Other: | ○ | ○ | ○ |
| Other: | ○ | ○ | ○ |

13. From the table in question 12, please name the three areas of ethical conflict situations that cause the greatest burden for your team:

No. _____

No. _____

No. _____

14. In what form does the ethics case consultation take place?

☐ specially convened team meeting for ethics consultation

☐ specially convened family conference for ethics consultation

☐ ethics rounds by an ethics consultant

☐ participation of an ethics consultant in routine meetings

☐ internal meeting because the ethics consultant is a team member

☐ telephone consultation

☐ online conference

☐ other: _______________________________

15. What are the three most positive effects you have experienced through ethics case consultation? (max. 3 answers, fewer are allowed):

☐ relief for those involved

☐ clearer definition of the problem

☐ greater confidence in difficult decisions

☐ more transparency in decisions

☐ better communication (e.g., within the team or with those affected)

☐ more respectful interactions

☐ feeling that the right decision was made

☐ conflicts could be resolved

☐ decisions are positively received

☐ the wishes of those affected can be implemented

☐ high motivation of participants in addressing the problem

☐ there are no positive effects

☐ other: ____________________________________

16. Please name the three biggest difficulties/obstacles in using ethics case consultation (max. 3 answers, fewer are allowed):

☐ low utilization despite existing need

☐ too time-consuming

☐ scheduling with all parties involved

☐ implementation of recommendations from ethics consultation

☐ lack of acceptance by attending physicians

☐ acceptance by relatives is difficult

☐ ethics case consultation is not desired in the institution

☐ limited knowledge about the fields of ethics consultation

☐ ethics case consultation is too little known overall

☐ meetings cannot be integrated into daily work schedule

☐ there are no problems

☐ other: _____________________________________

17. What improvements/innovations would you consider desirable for using ethics case consultations?

☐ higher level of awareness

☐ easier access

☐ ethics consultation via telephone

☐ “ethics consultation” hotline

☐ ethics consultation as tele-consultation (video conference)

☐ _____________________________________________

☐ _____________________________________________

18. Do you generally consider ethics case consultation useful? (please mark)

|_____|_____|_____|_____|_____|_____|_____|_____|_____|_____|
0 10
Not useful very useful

19. How high do you consider the need for ethics case consultation in your daily work? (please mark)

|_____|_____|_____|_____|_____|_____|_____|_____|_____|_____|
0 10
Not present very high

20. How satisfied are you with the ethics case consultation options currently available to you? (please mark)

|_____|_____|_____|_____|_____|_____|_____|_____|_____|_____|
0 10
not at all extremely

21. Would you like (more) support through ethics case consultation for your work?

☐ yes

☐ no

☐ unsure

Thank you very much for your participation!
