# Supplementary material for: Relevance of clinical ethics support services in specialized outpatient palliative care teams and inpatient hospices
Source: BMC Palliat Care. 2026 May 23;25:154. doi: 10.1186/s12904-026-02149-2 (PMC13198748; doi:10.1186/s12904-026-02149-2)
Supplement: Supplementary file 2 — Supplementary Material 2. [file 12904_2026_2149_MOESM2_ESM.pdf]

Fragebogen: Ethik-Fallberatung in deutschen Hospizen

1. Wer füllt den Fragebogen aus?
☐ gemeinsam als Team<br>☐ Mitarbeiter/-in der Pflege<br>☐ Arzt/Ärztin<br>☐ andere Berufsgruppe: ________________________________
2. Wie viele Mitarbeiter arbeiten in Ihrem Hospiz? (inkl. Vertragsärzt/innen, ehrenamtliche Helfer etc.)
☐ 1-10 ☐ 11-20 ☐ 21-30 ☐ 31-40 ☐ >40
3. Wie viele Patienten betreuen Sie durchschnittlich pro Jahr?
☐ 1-50 ☐ 51-100 ☐ 101-150 ☐ 151-200 ☐ >200
4. In welchem Bundesland liegt Ihr Hospiz?
____<br><br>Welche Wohnlage trifft auf Ihr Einzugsgebiet zu?<br>☐ Metropole (> 1 Mio. EW)<br>☐ Großstadt (> 100.000 EW)<br>☐ Mittelstadt (< 100.000 EW)<br>☐ Kleinstadt, ländlicher Raum (< 20.000 EW)
5. Kennen Sie Angebote der Ethik-Fallberatung?
☐ ja ☐ nein ☐ bin unsicher
6. Haben Sie schon einmal an einer Ethik-Fallberatung teilgenommen?
☐ ja ☐ nein ☐ bin unsicher
7. Haben Sie die Möglichkeit, Ethik-Fallberatung zu nutzen bzw. in Anspruch zu nehmen?
☐ ja ☐ nein ☐ bin unsicher

8. Nutzen Sie Ethik-Fallberatung in Ihrem aktuellen beruflichen Alltag?
ja nein bin unsicher<br><br>Wenn ja, wie oft: 0-5/Jahr 6-10/Jahr 11-20/Jahr >20/Jahr
9. Wenn Sie in Frage 8 „nein“ angekreuzt haben, können Sie uns die **drei wichtigsten Gründe** angeben, warum Sie Ethik-Fallberatung **nicht** nutzen?<br>(bitte **max. 3** ankreuzen, man darf auch weniger)
es gibt kein Angebot<br>bisher konnten wir alle Probleme ohne Hilfe lösen<br>eine Ethik-Fallberatung in Anspruch zu nehmen, ist im Alltag zu aufwendig<br>Ethik-Fallberatung erscheint uns nicht hilfreich<br>das Aufwand/Nutzen-Verhältnis ist zu schlecht<br>es gibt Widerstände im Team<br>das Hinzuziehen externer Personen wird nicht gewünscht<br>es ist schwierig, alle Beteiligten „an einen Tisch“ zu bekommen<br>Sonstiges: ____________________________<br><br>**Wenn Sie Frage 8 mit „Nein“ beantwortet haben, können Sie direkt zu Frage 16 auf Seite 5 springen**<br><br>**Wenn Sie mit „Ja“ geantwortet haben, machen Sie bitte hier (mit Frage 10) weiter**
10. Wie bearbeiten Sie ethische Fragestellungen der Alltagspraxis?
über ein eigenes Ethikkomitee<br>Ethikcafé (offenes Gesprächsforum)<br>interne Besprechungsrunden im Team<br>über ein (über-)regionales Ethikkomitee<br>über eine(n) ausgebildete(n) Ethikberater/in im Team selbst<br>über eine(n) ausgebildete(n) Ethikberater/in von außerhalb<br>über ein externes Ethikberatungs-Team (z.B. über die Ärztekammer)<br>wir lösen ethische Probleme über Therapiezielfindungsgespräche<br>sonstiges _____________________________________

11. Gibt es in Ihrem Team ausgebildete Ethikberater/innen (mindestens AEM* Stufe 1)

*AEM: Akademie für Ethik in der Medizin

□ ja □ nein □ bin unsicher

Wenn ja, wie viele? ____________

12. Bitte teilen Sie uns mit, wie häufig (nie, gelegentlich, oft) ethische Konfliktsituationen aus den unten aufgeführten Bereichen im Rahmen der Ethik-Fallberatungen in Ihrem Team eine Rolle gespielt haben:

	**Grund**	**nie**	**gel.**	**oft**
1.	Therapiebegrenzung (z.B. Beendigung einer med. Therapie oder Verlegung in ein Krankenhaus am Lebensende)	O	O	O
2.	Künstliche Ernährung/PEG (z.B. Beginn oder Beendigung)	O	O	O
3.	Umsetzung/Umgang mit der Patientenverfügung	O	O	O
4.	Ermittlung des mutmaßlichen Patientenwillens	O	O	O
5.	Einwilligungsfähigkeit von Patienten	O	O	O
6.	Recht auf Privatheit von Patienten	O	O	O
7.	Willensäußerungen bei dementen Patienten	O	O	O
8.	Patient/in verweigert medizinische oder pflegerische Hilfe	O	O	O
9.	Freiwilliger Verzicht auf Essen und Trinken	O	O	O
10.	Wunsch nach Assistenz beim Suizid	O	O	O
11.	Zwangsbehandlung von Patienten	O	O	O
12.	Freiheitsentziehende Maßnahmen (z.B. Fixierung)	O	O	O
13.	Gewalt gegen Personen oder Gegenstände	O	O	O
14.	Sexuelle Übergriffe	O	O	O
15.	Probleme mit Sexualität	O	O	O
16.	Interreligiöse oder –kulturelle Probleme	O	O	O
17.	Ethische Konflikte zwischen Angehörigen und Behandelnden	O	O	O
18.	Ethische Konflikte zwischen Angehörigen und Patient/in	O	O	O
19.	Ethische Konflikte im Behandlungsteam	O	O	O
20.	Sonstiges:	O	O	O
21.	Sonstiges:	O	O	O

13. Nennen Sie uns bitte (aus der Tabelle in Frage 12) die drei Bereiche ethischer Konfliktsituationen, die in Ihrem Team mit der größten Belastung einhergehen:
Nr._____<br><br>Nr._____<br><br>Nr._____

14. In welcher Form findet die Ethik-Fallberatung statt?
☐ einberufene Teambesprechung zur Ethik-Fallberatung<br>☐ einberufene Familienkonferenz zur Ethik-Fallberatung<br>☐ Ethikvisiten durch Ethikberater/in<br>☐ Teilnahme einer/s Ethikberater/in an Routine-Besprechungen<br>☐ interne Besprechung, weil Ethikberater/in Teammitglied ist<br>☐ Telefonische Beratung<br>☐ Online Konferenz<br>☐ sonstiges: ________________________________

15. Welches sind für Sie die **drei positivsten Effekte**, die Sie durch Ethik-Fallberatung kennengelernt haben? (bitte **max. 3** ankreuzen, man darf auch weniger)
☐ Entlastung der Beteiligten<br>☐ klareres Benennen des Problems<br>☐ größere Sicherheit bei schwierigen Entscheidungen<br>☐ mehr Transparenz von Entscheidungen<br>☐ bessere Kommunikation (z.B. im Team oder mit den Betroffenen)<br>☐ wertschätzender Umgang miteinander<br>☐ Gefühl die richtige Entscheidung getroffen zu haben<br>☐ Konflikte konnten gelöst werden<br>☐ Entscheidungen werden positiv aufgenommen<br>☐ die Wünsche der Betroffenen können umgesetzt werden<br>☐ hohe Motivation der Teilnehmer an der Bearbeitung des Problems<br>☐ es gibt keine positiven Effekte<br>☐ sonstiges: _____________________________________

16. Nennen Sie uns bitte die **drei größten Schwierigkeiten/Hindernisse** bei der Inanspruchnahme von Ethik-Fallberatung:
(bitte **max. 3** ankreuzen, man darf auch weniger)

- ☐ geringe Inanspruchnahme trotz bestehendem Bedarf
- ☐ zu aufwendig
- ☐ Terminfindung mit den Beteiligten
- ☐ Umsetzung der Empfehlung(en) aus der Ethikberatung
- ☐ Akzeptanz durch die behandelnden Ärzte nicht gegeben
- ☐ Akzeptanz durch die Angehörigen schwierig
- ☐ Ethik-Fallberatung ist in der Einrichtung nicht gewünscht
- ☐ geringe Kenntnis über die Aufgabenfelder der Ethikberatung
- ☐ Ethik-Fallberatung ist insgesamt zu wenig bekannt
- ☐ Besprechungen sind zeitlich nicht in den Arbeitsalltag einzubinden
- ☐ es gibt keine Probleme
- ☐ sonstiges: ____

17. Welche Verbesserungen/Innovationen halten Sie für die Inanspruchnahme von Ethik-Fallberatungen für wünschenswert?

- ☐ Höherer Bekanntheitsgrad
- ☐ unkomplizierte(re) Inanspruchnahme
- ☐ Ethikberatung in Form telefonischer Beratung
- ☐ „Ethikberatungs"- Hotline
- ☐ Ethikberatung als Tele-Beratung (Videokonferenz)
- ☐ ____
- ☐ ____

18. Halten Sie Ethik-Fallberatung generell für sinnvoll? (bitte Kreuz setzen)

|_____|_____|_____|_____|_____|_____|_____|_____|_____|_____|

0 Nicht sinnvoll — 10 sehr sinnvoll

19. Für wie hoch halten Sie den Bedarf an Ethik-Fallberatung in Ihrer täglichen Arbeit? (bitte Kreuz setzen)
|_____|_____|_____|_____|_____|_____|_____|_____|_____|_____| 0 ... 10   Nicht vorhanden ... sehr hoch
20. Wie zufrieden sind Sie bisher mit den Möglichkeiten der Ethik-Fallberatung, die Ihnen zur Verfügung stehen? (bitte Kreuz setzen)
|_____|_____|_____|_____|_____|_____|_____|_____|_____|_____| 0 ... 10   gar nicht ... außerordentlich
21. Würden Sie sich für Ihre Arbeit (mehr) Unterstützung durch eine Ethik-Fallberatung wünschen?
☐ ja ☐ nein ☐ bin unsicher

Herzlichen Dank für Ihre Teilnahme!
